# A Faithful Gut: Core Features of Gastrointestinal Microbiota of Long-Distance Migratory Bats Remain Stable despite Dietary Shifts Driving Differences in Specific Bacterial Taxa

**DOI:** 10.1128/Spectrum.01525-21

**Published:** 2021-11-24

**Authors:** Luis Víquez-R, Kelly Speer, Kerstin Wilhelm, Nancy Simmons, Rodrigo A. Medellín, Simone Sommer, Marco Tschapka

**Affiliations:** a Institute of Evolutionary Ecology and Conservation Genomics, Ulm University, Ulm, Germany; b Richard Gilder Graduate School, American Museum of Natural History, New York, New York, USA; c Center for Conservation Genomics, Smithsonian’s National Zoological Park & Conservation Biology Institute, Washington, DC, USA; d Department of Invertebrate Zoology, National Museum of Natural History, Washington, DC, USA; e Department of Mammalogy, Division of Vertebrate Zoology, American Museum of Natural History, New York, New York, USA; f Institute of Ecology, Universidad Nacional Autónoma de México, Mexico City, Mexico; g Smithsonian Tropical Research Institute, Panama City, Panama; Nanchang University

**Keywords:** 16S RNA, Chiroptera, gut microbiome, migration, Phyllostomidae

## Abstract

Migratory animals live in a world of constant change. Animals undergo many physiological changes preparing themselves for the migration. Although this field has been studied extensively over the last decades, we know relatively little about the seasonal changes that occur in the microbial communities that these animals carry in their guts. Here, we assessed the V4 region of the 16S rRNA high-throughput sequencing data as a proxy to estimate microbiome diversity of tequila bats from fecal pellets and evaluate how the natural process of migration shapes the microbiome composition and diversity. We collected samples from individual bats at two localities in the dry forest biome (Chamela and Coquimatlán) and one site at the endpoint of the migration in the Sonoran Desert (Pinacate). We found that the gut microbiome of the tequila bats is dominated largely by *Firmicutes* and *Proteobacteria*. Our data also provide insights on how microbiome diversity shifts at the same site in consecutive years. Our study has demonstrated that both locality and year-to-year variation contribute to shaping the composition, overall diversity, and “uniqueness” of the gut microbiome of migratory nectar-feeding female bats, with localities from the dry forest biome looking more like each other compared to those from the desert biome. In terms of beta diversity, our data show a stratified effect in which the samples’ locality was the strongest factor influencing the gut microbiome but with significant variation between consecutive years at the same locality.

**IMPORTANCE** Migratory animals live in a world of constant change. The whole-body ecosystem needs a strong adapting capacity to thrive despite the changes. Our study used next-generation sequencing to determine how gut microbial change along the migratory path of the nectar-feeding tequila bats. The study of the gut microbiome is a great tool that can provide important insights that are relevant not just for management and conservation but also an initial investigation of the extent of the adaptation and preparedness of the individual animals, with respect not just to their current environment but also to all the environments involved in their yearly cycle.

## INTRODUCTION

Migration is no easy task and often pushes animals to their physiological limits (1). Migratory animals exhibit many behavioral and physiological adaptations in order to prepare for and endure long migratory journeys. After arrival at a destination, the challenges are often not over, since long-distance migration can involve significant dietary changes and/or require adaptation to foraging in a new biome. These physiological burdens and dietary shifts affect not only the migratory animals themselves but also their gut microbiomes, which play a central role in food processing and synthesizing vital nutrients (2). Although migratory animals are often assumed to depend solely on their own energetic resources to survive and thrive during and after migration, their faithful microbial travel companions may also play an essential role in enabling animals to endure the stresses of migration.

Many studies indicate that the taxonomic and functional composition of mammal gut microbiomes is driven primarily by diet and host phylogeny (see reviews 3 and 4). Indeed, a recent comparative meta-analysis of mammalian and bird gut microbiomes has shown that, within most terrestrial mammal orders, strong correlations exist between microbial community composition and host diet, and the phylosymbiotic signal is strong (5). However, in flying vertebrate groups, such as bats and birds, host-specific microbiomes exhibit a lower phylosymbiotic signal than expected (6, 7), suggesting that host-gut microbiome phylosymbiosis might be convergently affected by factors associated with the physiological adaptations to flight. However, Ingala et al. established that, within bats, the different dietary “guilds” show different bacterial enrichment (8).

If the evolution of powered flight is a strong force in shaping the microbiome, what additional impact on the microbiome occurs in migratory species as they move across biomes and undergo shifts in diet? More specifically, do microbiome diversity and composition remain unchanged before and after migration, or are they influenced by diet regime changes associated with the different biomes, or both? Studies of the microbiome of migratory animals are limited and are restricted mostly to birds (9–11), with sparse information being available about bats.

Within the more than 1,430 bat species known currently (12), only 87 species migrate (13), and fewer than 30 of those species undergo long-distance (more than 500 km) migrations (14). Among the last-mentioned group, we find the tequila bat (Phyllostomidae: Leptonycteris yerbabuenae), a nectar-feeding species in which some members of the population embark every year on an exceptional journey. Between October and February, both males and females overwinter in a series of caves in the Pacific dry forests of Mexico (15, 16). In early March, pregnant females leave their wintering sites to begin a migration of ∼1,000 km to the Sonoran Desert in the north of Mexico (17, 18), whereas males and some females stay behind (16, 19). This nectar-feeding bat has a broad diet in the dry forest biome of southern Mexico, feeding from flowers of *Ipomoea* spp., a wide variety of columnar cacti, *Agave* spp., Cleome spinosa, Ceiba pentandra, and other Malvaceae (20). In contrast, the diet of L. yerbabuenae in the desert biome of the Sonoran Desert is restricted to a few flowering columnar cacti species (16).

In this study, we test the hypothesis that the dietary shifts experienced during migration alter the microbiome of tequila bats, with the microbiome becoming narrower in the desert and more diverse in the dry forest. Consistent with previous studies, we expected to observe a bird-like microbiome in this bat species as has been shown for other bats (5). If the evolution of powered flight is the primary force in shaping the microbiome, we would expect only minor differences in the diversity and composition of the microbiome of *Leptonycteris yerbabuenae* before and after migration. However, if adaptations also occur with regard to the dietary changes at both ends of the annual migration, we would expect locality-specific signals. In particular, since female bats return every year from a variety of localities in the diet-rich southern dry forest biome to the Pinacate in the Sonoran Desert where they are restricted to a columnar cactus-based diet, the microbiome of bats in the desert biome should show a lower alpha diversity than in the dry forest localities, given the more restrictive diet, but a higher annual variation in beta diversity in consecutive years because the year-to-year input from the incoming individuals changes. An understanding of the impact of migration and its associated diet changes on the composition and diversity of the microbiome is crucial for the development of conservation strategies that focus on preserving not only numbers of individuals within a species but healthy and competent individuals well suited to the natural habitats that they visit throughout the year.

## RESULTS

We collected fecal samples from 520 individual bats (dry forest biome, *n *= 307; desert biome, *n *= 213; Table S2). Following 16S rRNA sequencing, we obtained ca. 34 million reads, of which 14 million reads covering 12,200 amplicon sequence variants (ASVs) were kept after filtering and denoising (DADA2) and after chloroplast and mitochondria removal. Once those samples containing fewer than 8,000 reads had been filtered out, we retained 487 samples distributed between Chamela, Coquimatlán, and Pinacate. The mean coverage for each sample was 31,260 (± 12,550), ranging between 8,367 to 65,000 reads per sample; a total of 11,926 ASVs were identified.

### Tequila bats show a “bird-like” microbiome that harbors different taxa across biomes.

**FIG 1:**
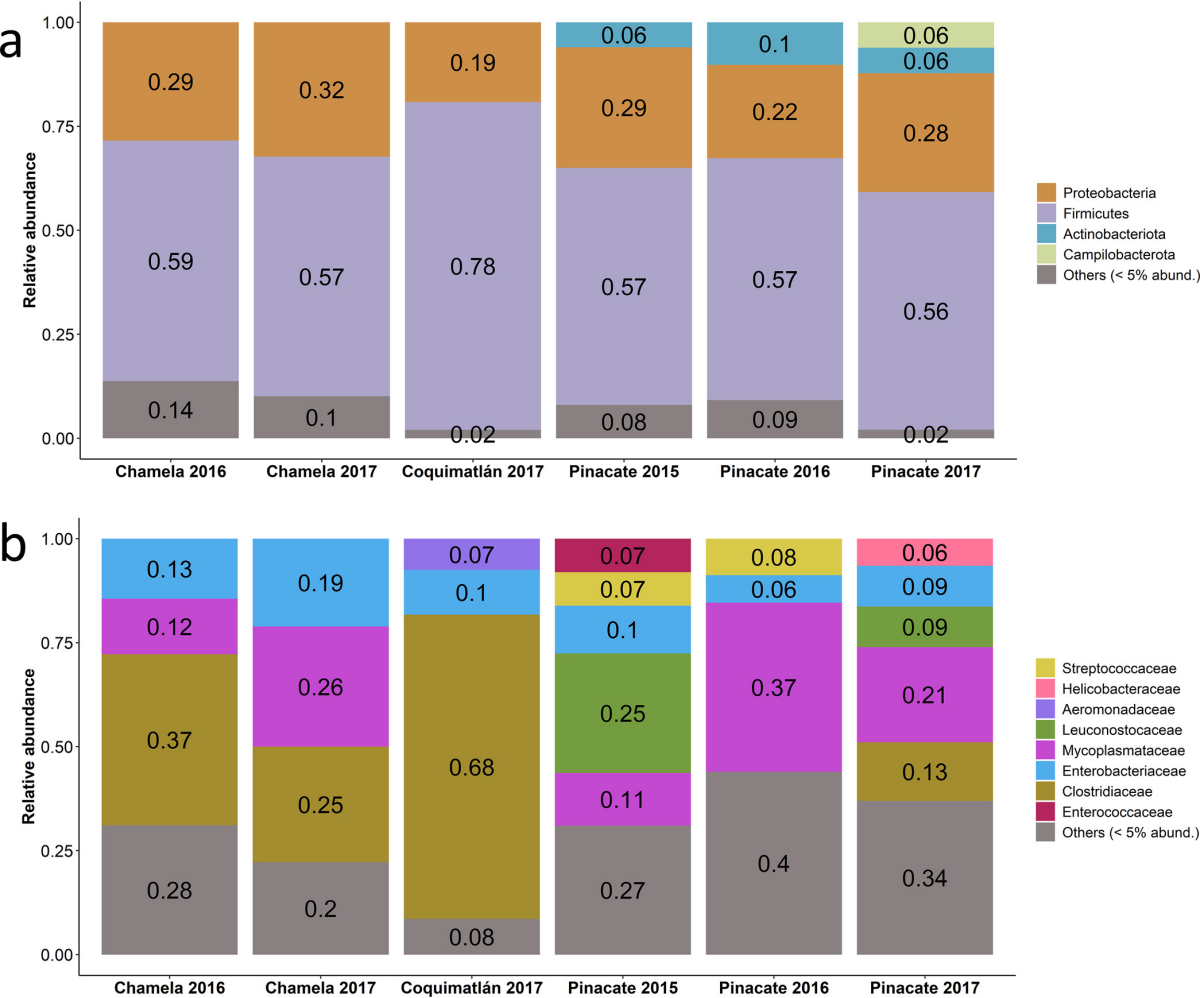
Composition of the gut microbiomes of tequila bats (*Leptonycteris yerbabuenae*) in the dry forest (Chamela 2016 to 2017; Coquimatlán 2017) and in the desert (Pinacate 2015 to 2017) biomes. Relative abundance of (a) major phyla and (b) families.

Consistent with previous studies comparing volant vertebrates to nonvolant relatives (3), the gut microbiome of *Leptonycteris yerbabuenae* revealed very low diversity levels compared with those of other nonflying mammals. The microbiome was dominated mainly by the phyla *Firmicutes* (56 to 66%) and *Proteobacteria* (25 to 30%), with minor contributions from *Actinobacteriota* (Fig. 1a). At the family level, the microbiome of bats from different localities revealed variation in composition depending on whether the bats were in the dry forest or desert biome; significant variation was also observed between years (Fig. 1b). Variation between years was particularly evident in the bats from the Pinacate locality in the desert biome. The relative abundance of *Leuconostocaceae* varied between years, ranging from 25% in 2015 to less than 5% in 2016 and then increasing to 9% in 2017. Concomitantly, the relative abundance of *Mycoplasmataceae* and *Enterobacteriaceae* also varied considerably among the different years at this site (Fig. 1b).

### Microbiome alpha diversity shows a locality-specific response even within the same biome.

**FIG 2:**
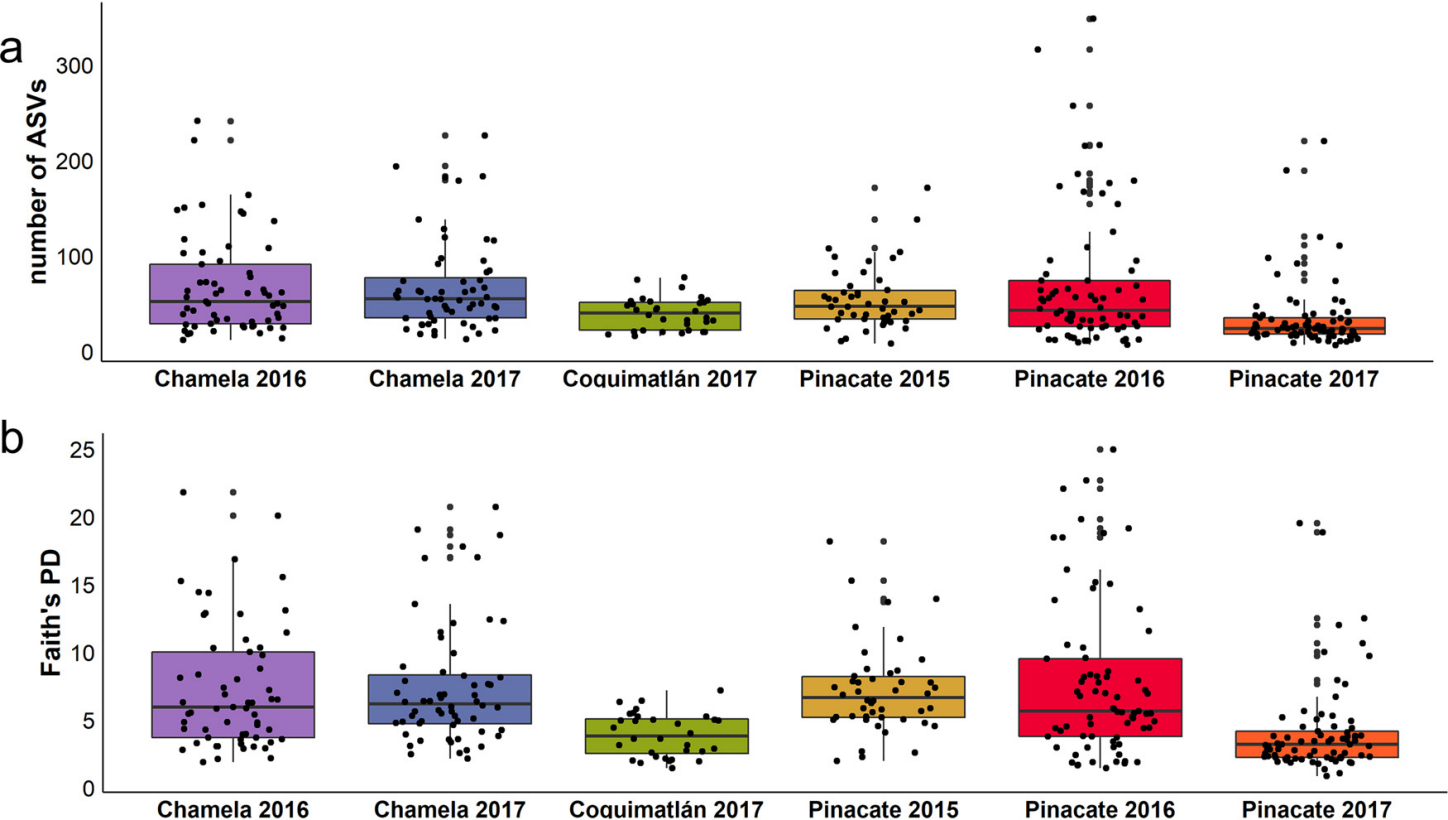
Gut microbial alpha diversity in bats captured within the dry forest (Chamela 2016 to 2017; Coquimatlán 2017) and desert (Pinacate 2015 to 2017) biomes. (a) Taxa richness (ASVs) and (b) Faith’s phylogenetic diversity.

Although taxa richness (ASV) and the Shannon-Wiener diversity were not significantly different between biomes, the microbiome phylogenetic diversity (Faith’s PD: *P *= 0.03) was higher in the dry forest bats than in the desert bats (Fig. S1A, Table S3), indicating the presence of additional microbial taxa. Alpha diversity differed between localities and between years, for both the ASVs (locality, *P* < 0.001; year, *P* < 0.001) and Faith’s phylogenetic diversity (locality, *P* = 0.001; year, *P* = 0.02), whereas Shannon-Wiener diversity remained unchanged (locality, *P* = 0.09; year-to-year changes, *P* = 0.8) (Fig. S1B, Table S3). This indicates that, although the overall community entropy remains the same (Shannon-Wiener) across individuals from all localities, the number of individual taxa (ASVs) and the phylogenetic breadth of individual microbiome (Faith’s phylogenetic diversity) are heavily influenced by the locality. Pairwise comparisons (Tukey’s honestly significant difference test [Tukey’s HSD test]) showed stratified differences between localities (ASVs, *P* < 0.05; Faith’s phylogenetic distance [FPD], *P* < 0.001) and between years, with 2015 and 2016 not being different from each other but both always being different from 2017 (Fig. 2).

### Beta diversity is driven by both locality and year-to-year variation and is most extreme in the desert biome.

**FIG 3:**
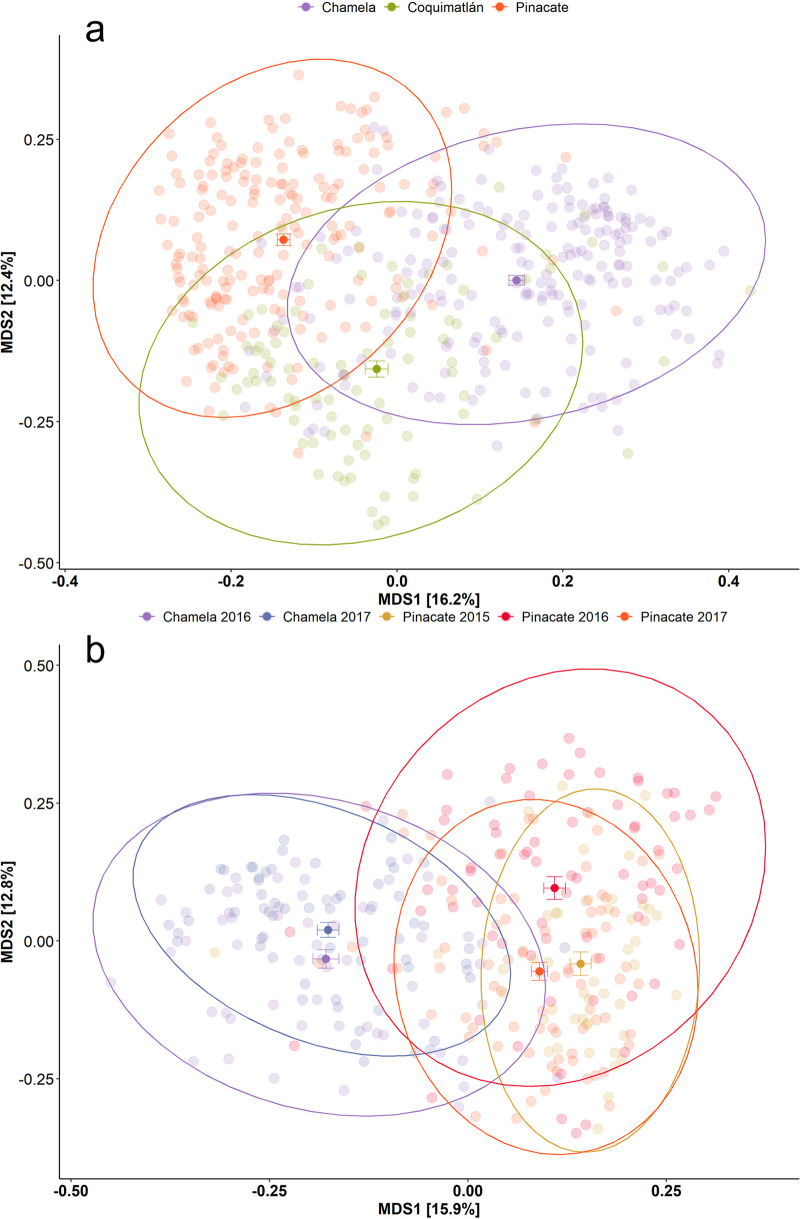
Multidimensional scaling (MDS) of unweighted UniFrac distances between the gut microbiomes of *L. yerbabuenae* individuals. Samples are color-coded by locality (a) and by year-locality arrays (b). The centroids for each grouping are indicated with their standard error. The dry forest biome at the Pacific coast is exemplified by Chamela and Coquimatlán, whereas the desert biome is represented by Pinacate.

**FIG 4:**
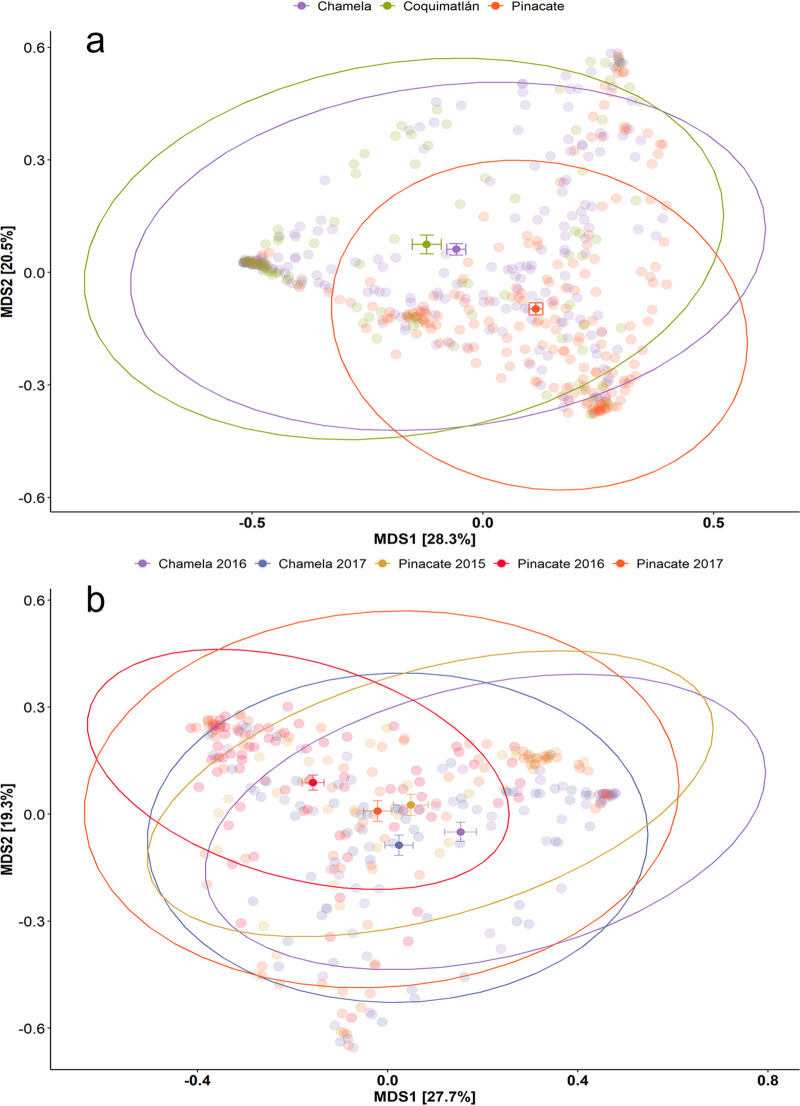
Multidimensional scaling (MDS) of unweighted UniFrac distances between the gut microbiomes of *L. yerbabuenae* individuals. Samples are color-coded by locality (a) and by year-locality arrays (b). The centroids for each grouping are indicated with their standard error. The dry forest biome at the Pacific coast is exemplified by Chamela and Coquimatlán, whereas the desert biome is represented by Pinacate.

Beta diversity shows a striking pattern for the unweighted (Fig. 3) and weighted UniFrac indexes (Fig. 4). Gut microbiomes disperse neatly around the centroid for each locality, with the ellipsis showing an equal distribution over the multidimensional space. This effect is even more evident in the unweighted UniFrac, where samples cluster by locality (Fig. 3a) or year-locality arrays (Fig. 3b). The permutational multivariate analysis of variance (PERMANOVA) confirms these patterns, as the locality is the strongest explaining factor for the unweighted UniFrac (R2 = 0.15, *P* < 0.001) (see Table S4a and b in the supplemental material).

The same effect appears in the weighted UniFrac (Fig. 4a) index (R2 = 0.087, *P* < 0.001) but with a lower goodness of fit. However, in this case, the centroids for both dry forest localities are closer to each other than to Pinacate. Considering taxonomic breadth and relative abundance (weighted UniFrac), individuals from the dry forest biome are closer to each other than to the individuals from the desert biome. This pattern is also supported by the effect size analysis (Cohen’s D). When we compared the microbiomes of the Coquimatlán and Chamela bats, we found no difference between those groups (Table 1). All of the unweighted UniFrac Cohen’s D coefficients for pairwise comparisons between all localities have high values (>1), indicating a large effect. Hence, the microbiome’s core features appear to be most strongly associated with the biome, whereas the low-abundance features (better represented by unweighted UniFrac) are better explained by the locality (Table 2).

**TABLE 1:** Cohen’s D values for weighted UniFrac distance comparisons between localities (2015 to 2017)

Locality	Value for:		
	Chamela	Coquimatlán	Pinacate
Chamela		[−0.12, 0.30]	[0.50, 0.91]
Coquimatlán	0.1292		[0.59, 1.11]
Pinacate	0.7032	0.8486	

**TABLE 2:** Cohen’s D values for unweighted UniFrac distance comparisons between localities (2015 to 2017)

Locality	Value for:		
	Chamela	Coquimatlán	Pinacate
Chamela		[0.87, 1.40]	[1.31, 1.76]
Coquimatlán	1.1369		[1.09, 1.63]
Pinacate	1.5329	1.3607	

Beta diversity shows a specific pattern, where the differences between the year-locality arrays are significantly different for both the weighted and unweighted UniFrac (Table 3 and 4). For the weighted UniFrac, a clear association was observed by locality. Year-to-year variation was the smallest in the locality of Chamela (purple and blue), where the centroids were closest to each other (Fig. 3b). In Pinacate, we see that the year-to-year variation is greater, with centroids being more distinctly separated and exhibiting greater data dispersion for 2017 (Tukey HSD, *P* < 0.001; see Table S4 in the supplemental material). The weighted UniFrac shows a similar pattern for the locality and biome results wherein all centroids are close to each other, except for the Pinacate data in 2016, which are shifted slightly to the upper right in the plot (Fig. 4b). In a nutshell, we again see that rare and low abundant taxa (unweighted UniFrac) drive the beta diversity differences between the arrays, with more data dispersion for Pinacate between and within years. Simultaneously, the core features of the microbiome (better represented by the weighted UniFrac) remain similar, even when bats change their diet drastically.

**TABLE 3:** PERMANOVA results for the year-to-year comparison of weighted UniFrac distances between Chamela (2016 to 2017) and Pinacate (2015 to 2017)

Characteristic	Df^*a*^	SumsOfSqs	MeanSqs	F.Model	R2	Pr(>F)
Arrays	4	10.110	2.52741	10.425	0.12	<0.0001
Residuals	312	75.641	0.24244		0.88	
Total	316	85.751			1.00	

a Df, degrees of freedom; Pr, probability.

**TABLE 4:** PERMANOVA results for the year-to-year comparison of unweighted UniFrac distances between Chamela (2016 to 2017) and Pinacate (2015 to 2017)

Characteristic	Df	SumsOfSqs	MeanSqs	F.Model	R2	Pr(>F)
Arrays	4	9.757	2.43913	15.641	0.17	<0.0001
Residuals	312	48.654	0.15594		0.83	
Total	316	58.410			1.00	

### Gut microbiomes of dry forest- and desert biome-foraging bats harbor a differential abundance of specific ASVs.

Differences are not just restricted to the presence or absence of specific microbial taxa, as the abundance of some of these rare taxa differs distinctly between the biomes. The differential abundance analysis (ANCOM) shows that 17 ASVs are differentially abundant between biomes and localities, with the most considerable differences being observed between biomes (right) and less-striking differences being observed within the dry forest biome (Chamela and Coquimatlán) (Fig. 7). This is evident from the center log-ratio values (CLR), where few ASVs showed a significant divergence between localities. The genus *Sarcina* offered a good summary of the general trend: the abundance of this ASV is higher in the dry forest biome than in the desert biome. No differential abundance is seen in *Sarcina* in a comparison of the two dry forest localities (Coquimatlán and Chamela). Our results also show that the genus *Streptococcus* is more abundant in the desert biome than in the dry forest biome. Nevertheless, a comparison of the abundance between the two dry forest localities shows no differences.

## DISCUSSION

One of the most significant challenges for any organism is to maintain homeostasis. Alternative body plans and physiologies have evolved repeatedly to maintain equilibrium in temperature (21), glucose levels (22), and fluid balance (23). The problem of homeostasis becomes particularly interesting in the case of the microbiome. Individual vertebrate hosts rely on the microorganisms of the microbiome for conducting normal processes, such as fermentation (24) and vitamin absorbance (2), and this inner ecosystem is also part of the early warning system for infection. One example of the latter is the Peyer’s patches, in which immunocytes constantly survey the bacteria in the ileum (25). Therefore, an adaptive advantage exists in the facilitation of rapid microbiome turnover, which thus enables a faster adaptation to food resources and their associated bacteria while allowing the early warning system to remain fully functional (26, 27). However, the hosts are walking on a tightrope. A quick turnover of gut microbiomes can also have potential downsides, as high susceptibility to environmental changes might in some cases shift the microbiome rapidly toward dysbiosis (28).

Community composition and alpha diversity patterns of the microbiome of *Leptonycteris yerbabuenae* largely mirror those found in other studies of bat microbiomes (8, 29, 30). The microbiome of the tequila bats is dominated by a large proportion of *Proteobacteria* and *Firmicutes*, as is common for bats (4). Song et al. (5) have proposed that evolution of powered flight is a vital shaping force for the microbiome of flying vertebrates. Our results thus confirm such a “bird-like” microbiome pattern for the tequila bat.

Our study demonstrates that *Leptonycteris yerbabuenae* has a distinct microbiome shaped by both geographic turnover (i.e., biome-specific signals) and dietary differences. Microbial alpha diversity shows a locality-specific response even within the same biome. Bat microbiome samples taken within the dry forest showed a higher taxonomic richness and a wider phylogenetic breadth than the samples from bats living in the desert. A more varied diet is thus seemingly linked to a more diverse microbiome, suggesting that the dry forest offers a more diverse range of dietary resources (and microbial sources) than the desert, where the diet comes merely from a few cactus species (31, 32). The differences observed between the bat microbiomes of the dry forest suggest that, despite a high similarity in local food resource availability (32), bats still vary in local food preferences. We hypothesized that adaptations to dietary changes at both ends of the migration would affect the microbiome and that locality-specific signals would be present in our data set. Our results confirm this hypothesis, as the dry forest sites, where the diet is more diverse (18), showed also a higher gut microbiome diversity. We expected that different biomes would support different microbiome communities and that beta diversity differences between biomes and localities would exhibit a nested effect, i.e., microbiome composition in Chamela and Coquimatlán would be more similar to each other than to Pinacate. Our data confirmed this expectation, with the gastrointestinal microbiome of bats from the two dry forest localities (Chamela and Coquimatlán) sharing more taxa and showing more similar structure compared with the microbiome of bats from the northern desert at Pinacate. Our data suggest that microbiome composition and diversity are shaped by both the migration process and the dietary shifts along the migration. However, we can provide only inference to both processes since our data come from one site in the desert and just a couple of sites in the dry forest. Since both factors work in synergy, it would be important for new studies to add multiple sampling sites in both biomes and collect diet samples to be able to disentangle the effect of diet and migration.

The ANCOM analysis demonstrated that the bacteria genus *Sarcina* was highly differentially abundant between samples from the dry forest and those from desert biomes (Fig. 3). This genus belongs to the *Clostridiaceae*, a bacterial family commonly found in mammals. In Sierra Leone, this genus has recently been linked to a disease known as epizootic neurologic and gastroenteric syndrome (ENGS) in chimpanzees (33). Over 13 years, 56 animals died from ENGS in the animal sanctuary where the study took place. A postmortem analysis demonstrated that the bacteria were found not only in the feces but also in the brain, liver, and spleen of the chimpanzees. The genus has also been reported in bat species such as Plecotus auritus in the United Kingdom (34) and Rousettus amplexicaudatus in the Philippines (25). Nevertheless, no adverse health conditions have been reported in bats. Further studies and dedicated sampling are needed to assess whether *Sarcina* or any other *Clostridiaceae* pose any health risks for bats.

Another highly variable genus was *Streptococcus*, which was more abundant in samples from the desert than in those from the dry forest biome. This is a vast genus, with more than 75 reported species (35). *Streptococcus* species lack many biosynthetic enzymes for essential amino acids and require other commensal bacteria to fulfill their requirements (36). Most species are facultative pathogens, with the notable exception of Streptococcus thermophilus (37), and some confirmed pathogenic species (i.e., Streptococcus pyogenes and Streptococcus pneumoniae) are also often found in healthy humans. *Streptococcus* spp. are common elements of the mammalian microbiome, and some species have been described from the oral cavity of bats (38). Reese et al. (39) found that *Streptococcus* is more common in the early life stages of wild chimpanzees than later in life. The Pinacate cave is the largest maternity roost known for the tequila bat (19), and the high prevalence of *Streptococcus* spp. might be explained by the constant presence of immuno-naive newborn bat pups (39).

**FIG 5:**
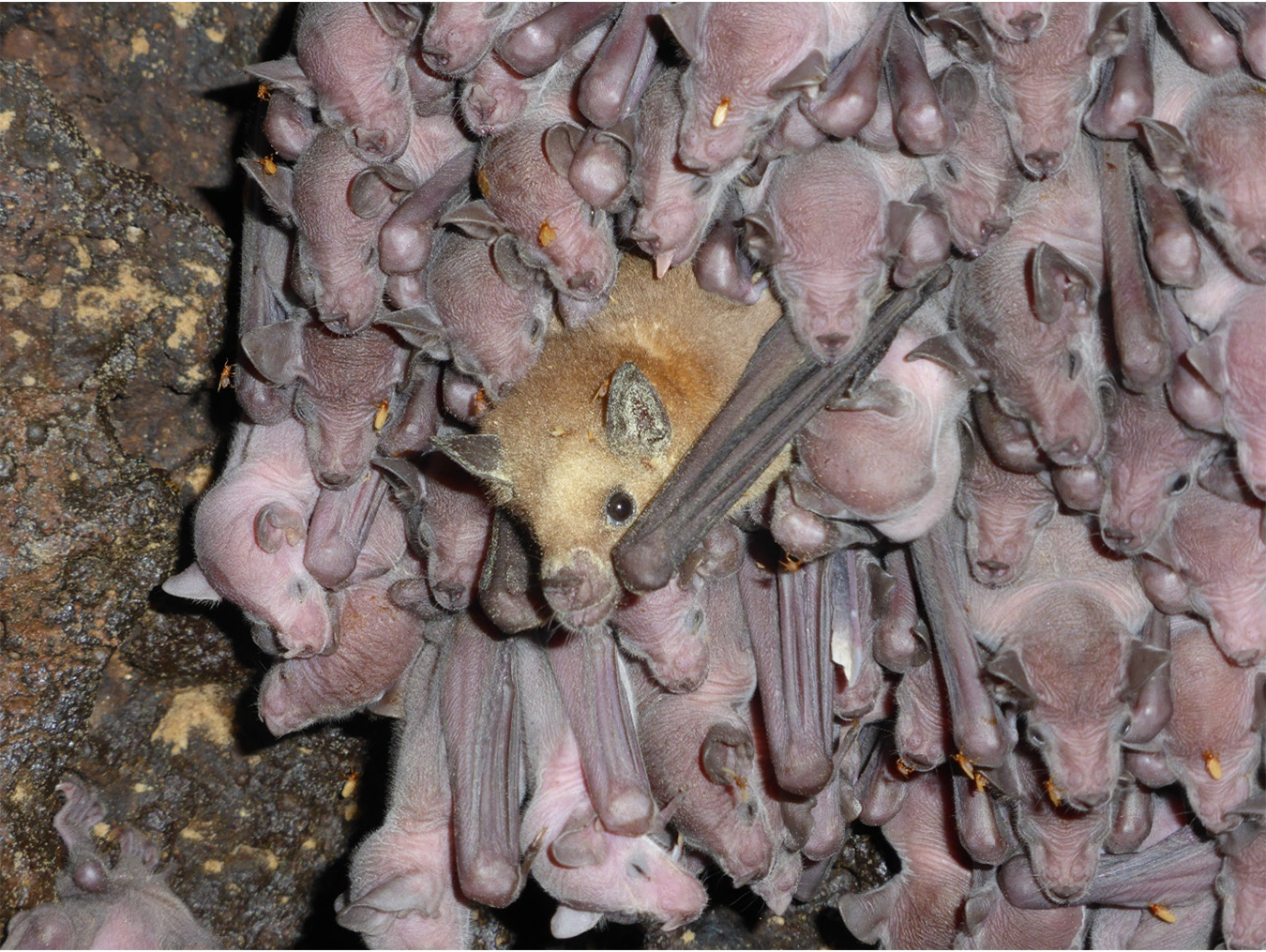
Adult female on the edge of a nursery patch in the Pinacate cave. The orange flies are bat flies (Streblidae).

In early May, shortly after the migrant females arrive in the Sonoran Desert, they give birth to their pups, and the cave population almost doubles in size (16). During the first couple of weeks, while the pups are still nonvolant, the mothers cluster the pups together into large nurseries (∼200 pups) in the hottest parts of the cave (40). While the females are out foraging during the first half of the night, the pups are tightly clustered in close proximity to each other. They are constantly licking, smelling, and biting each other within these tight aggregations, while also urinating and defecating (40). When the females come back from their nightly foraging trips, they land within this tight aggregation and start searching for their pup (Fig. 5). Although the pups have not changed their location during the night, the females still have to search for their own offspring and interact with many pups in this process, smelling and licking both the heads and genitals of many pups (40). This intense interaction among individuals, in conjunction with the more restrictive diet, may constitute a critical homogenization factor for the gastrointestinal microbiome of the pups and consequently might also be a strong influence on the microbiome of the females.

Resource sharing is another important factor that may contribute to the microbiome homogenization in nectar-feeding bats. Flowers are communal resources that may be shared by many individuals in the same night (41). After a bat has visited and drunk from the flower, the nectar and the flower microbiome might progressively change, given the inadvertent microbiome donations from the pollinators. In Pinacate, some bats visit the same flowers on any given night, which could also be a robust homogenizing force for the microbiome of the tequila bats, especially in the northern part of their distribution where the flower patches are aggregated, with animals spending several hours within a small territory visiting all the flowers in a patch (31).

### Conclusions.

Our study has demonstrated that both locality and year-to-year variation contribute to shaping the composition, overall diversity, and uniqueness of the gut microbiome of migratory nectar-feeding female bats. Both factors work in synergy, and isolation of their individual contribution is complicated. In general, patterns emerge at the locality level, although a single year might not represent the overall condition or state of the microbiome for a region. Our investigation highlights the importance of including multiyear sampling in microbiome research, and additionally researchers need to account for geographical and temporal differences in their studies. Future work should explore the possible importance of pregnancy and lactation in the vertical transference of the gut microbiome. The birth boom in Pinacate is a perfect opportunity for studying the development of the microbiome in early life and the influence that the communal microbiome might have in shaping this development. Study of the gut microbiome is a great tool that can provide (i) important insights that are relevant for management and conservation and (ii) an inside look into the extent of the adaptation and preparedness of the individual animals with respect not just to their current environment but also to all the environments involved in their yearly cycle.

## MATERIALS AND METHODS

### Sample collection.

**FIG 6:**
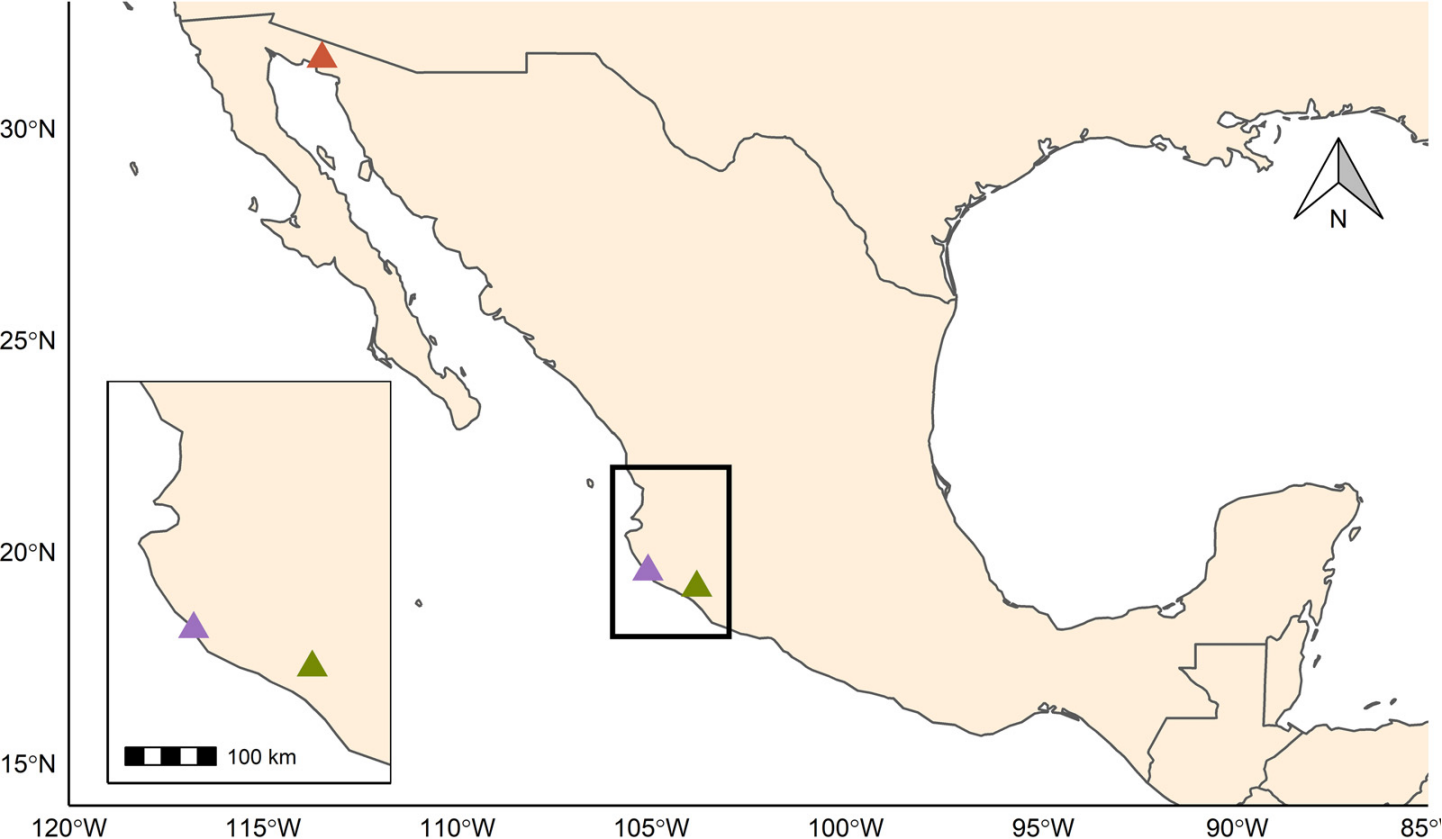
Sampling localities in Mexico. Sampling took place in two different biomes: in the dry forest biome at the Pacific coast (localities Chamela [purple] and Coquimatlán [green]) and in the desert biome (locality Pinacate [orange]), located about 1,000 km north of the first biome area.

**FIG 7:**
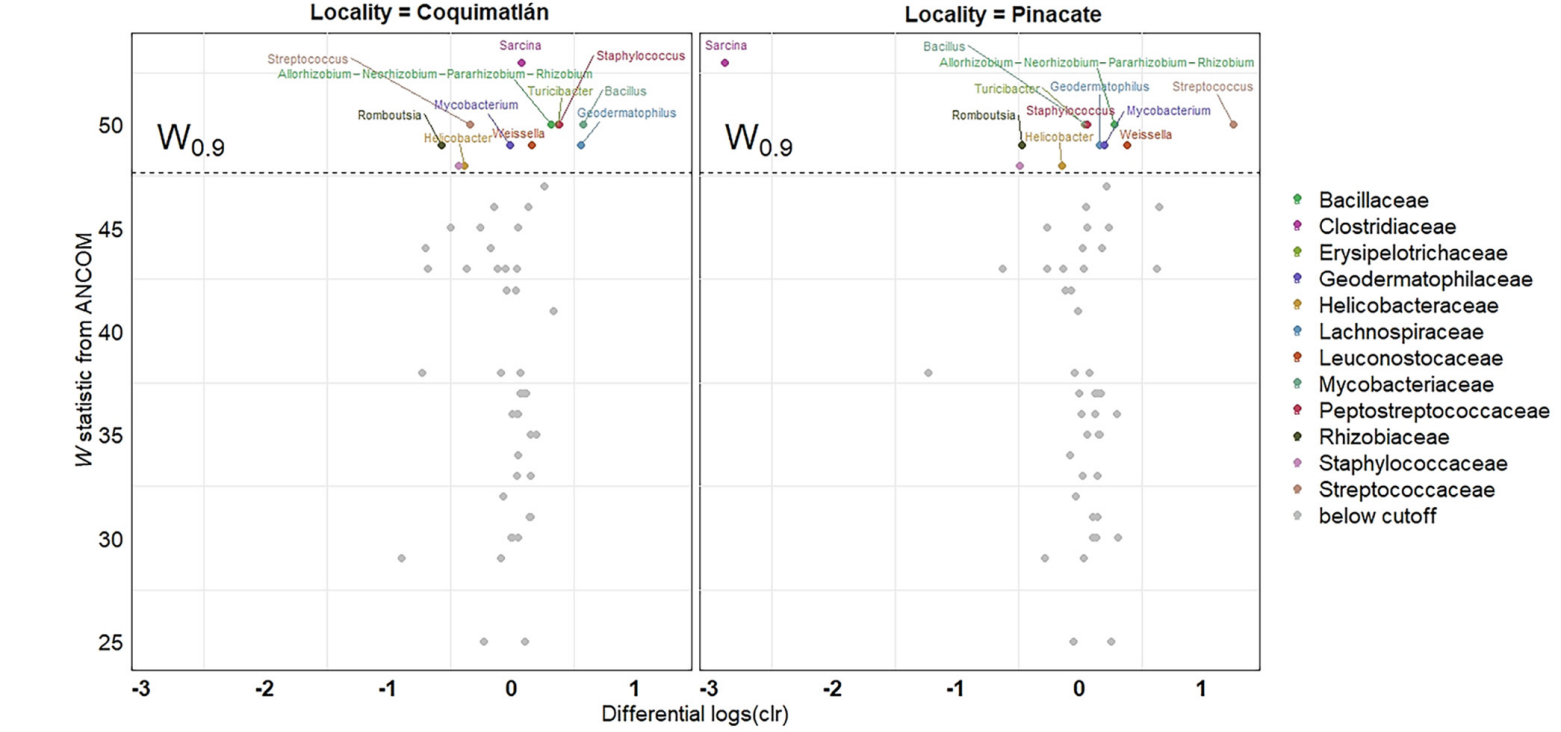
Differential abundance of taxa (center log ratios) observed in the gut microbiomes of bats captured in the dry forest biome (Chamela versus Coquimatlán) (left) and in the dry forest and desert biomes (Chamela versus Pinacate) (right). ASVs are colored by family only when the cutoff W value of 0.9 was met. Each differentially abundant ASV is labeled according to the genus.

Between 2015 and 2017, we captured *Leptonycteris yerbabuenae* returning from their nightly foraging trip in close vicinity to roost sites at both ends of their annual migration (i) during November in the overwintering area within the Pacific Dry Forest of Mexico (Dry Forest biome), specifically on Don Panchito Island (19°32′03.8′′N 105°05′17.7′′W) off the coast of the Chamela-Cuixmala Biosphere Reserve, State of Jalisco (hereafter Chamela), and in La Fábrica mine (19°09′04.2′′N 103°50′05.3′′W) in the town of Coquimatlán, State of Colima (hereafter Coquimatlán), and (ii) during early May in the largest known maternity cave for this species, namely, the Pinacate cave (31°38′51.4′′N 113°28′51.2′′W) (hereafter Pinacate) within the Pinacate and Gran Desierto de Altar Biosphere Reserve in Northern Sonora, Mexico (Desert Biome) (18, 42, 43) (Fig. 6 and 7). Details of the localities are provided in Table S1.

At Chamela and Pinacate, we captured bats in mist nests (dimensions: 12 by 2.5 m, mesh: 16 mm, Ecotone, Poland) positioned near the cave entrance. In Coquimatlán, we captured the animals by using a hand net (18-in diameter Polyester bag, Tropics Net, BioQuip, USA) attached to a 3.6-m extension pole. Upon capture, bats were removed from the net and placed into a clean soft-cloth bag until processing (<60 min). We followed the guidelines of the American Society of Mammalogists for safe and humane animal care throughout our study (44). We collected data concerning the body mass, forearm length, approximate age, and reproductive status for each individual captured. Bats were divided into two age categories (adults and juveniles) based on the degree of epiphyseal-diaphyseal ossification and fusion at joints in the wing (45). During this study, we focused our fecal sampling on adults only. For reproductive status, we classified females into three categories, pregnant, lactating, or reproductively inactive, based on abdominal distension, palpation of the abdomen, hair loss around the nipple, and milk secretion, while males were divided into active or inactive depending on whether the testicles had descended (reproductively active) or were abdominal (inactive). We encountered only pregnant females in Pinacate. Out of 174 captured females in the dry forest biome, only two were pregnant; we excluded these females in order to focus on the patterns that we consider to represent the migrating population of females that mate in late winter and then migrate to the desert in spring. We collected a single fecal pellet from each individual and stored it in a safe-lock 1.5-mL Eppendorf tube (Eppendorf, Germany) containing 500 μL DNA/RNA shield buffer (Zymo Research Europe GmbH, Germany). We inverted the tube several times to ensure maximum impregnation of the sample and stored in a cooler until frozen at −20°C.

### DNA extraction, amplification, and 16S rRNA sequencing.

We followed the Earth Microbiome Protocol (46) as modified by Víquez-R et al. (47) to include PNA-DNA clamps to block the signal of the rRNA of chloroplasts and mitochondria from the diet of the bats in the desert samples. We extracted the samples (in randomized sampling order) using the NucleoSpin soil extraction kit (Macherey-Nagel, Düren, Germany). We amplified a 291-bp fragment of the V4 region of the 16S rRNA gene by using the Earth Microbiome primer set 515F and 806R (46) with tagged (CS) target-specific (TS) primers: CS1-NNNN-TS-515F and CS2-TS-806R. We added four random bases to our forward primers to facilitate cluster identification during the first cycles of the Illumina MiSeq System (Illumina, San Diego, USA). During the first amplification step, we added cpPNA (47) and mPNA (48) to give a final concentration of 1 μM in order to reduce the effect of the high level of plastids and mitochondria coming from the diet in the desert (both chloroplast and mitochondria) and from the dry forest (only mitochondria). In the second step, we used the CS tags to add 10-bp barcodes to all the samples to multiplex in the sequencer. We included negative controls in the extraction and both amplification steps. The NucleoMag NGS clean-up and size select kit (Macherey-Nagel, Düren, Germany) was employed in a GeneTheatre (Analytik Jena, Jena, Germany) to clean the PCR products according to the manufacturer’s guidelines. We measured the quality of the amplicons by using the QIAxcel advanced system (Qiagen, Hilden, Germany) and quantified the DNA concentration by means of the PicoGreen QuantiFluor dsDNA system (Promega, Madison, USA) on a TECAN Infinite F200 PRO plate reader (Tecan, Männedorf, Switzerland). We included 20 ng of purified DNA of each sample in the final library; this library was then diluted down to 3 nM and spiked with 5% PhiX sequencing control V3 (Illumina, San Diego, USA). We performed paired-end sequencing of the amplicons following the manufacturer’s instructions (Illumina, MiSeq reagent kit v2—Reagent Preparation Guide) and loaded a final library with a concentration of 6 pM. Sequencing consisted of paired-end sequencing over two × 250 cycles (see Víquez-R et al. [47] for more details). All the raw sequencing data are deposited in Dryad.

### Demultiplexing and quality-filtering.

We conducted all the initial processing (demultiplexing and denoising) using the QIIME2 CLE pipeline (version 2020.2 [43]) in a Linux Mint 19.2 environment. The DADA2 method was used for chimera and artifact filtering (49). We trimmed the reads at 200 bp (mean quality score: 37) in both directions. Subsequently, we assigned the taxonomy to each amplicon sequence variant (ASV) by using a SILVA V4 classifier (SSU release 138 515806) object with the “*qiime feature-classifier classify-sklearn*” function in QIIME2 at the highest level of taxonomical resolution (level 7). We then filtered out the ASVs recognized as chloroplast, mitochondria, and archaea. We regenerated the filtered feature table and the representative sequences objects for further analysis. Finally, we imported the final objects into R (version 3.6.2 [50]) by using the *phyloseq* package (51). We deposited all scripts in the project site on Github (https://github.com/luisvqz/tequilabat).

### Statistical analysis.

We preprocessed our data by removing taxa identified in the extraction and PCR controls (blanks) from the samples. Subsequently, we removed any sample with fewer than 8,000 reads from the data set. We rarefied our data set to 8,300 reads for all samples for all consequent analyses (both alpha and beta diversity). We “melted” our database into either family or phylum level. The relative abundance of any specific taxa in the samples at the phylum and family level was calculated according to biome and to sampling locality. We filtered all the taxa that represented less than 5% of all reads and pooled them into a new category called “Others 5%.” This allowed us to compare the abundant features of the microbiome in compositional plots on various taxonomical levels.

For alpha diversity measures, we used the number of amplicon sequence variants (ASVs) as a proxy for taxon richness (52), the Shannon-Wiener index to determine the community entropy, and finally Faith’s phylogenetic diversity (Faith’s PD) index (53), which considers not only the diversity and evenness but also the phylogenetic distances between taxa. These metrics were calculated for each sample since each individual represents a uniquely assembled bacterial community. We normalized the alpha diversity measures by using a square root transformation for the Shannon-Wiener index and a logarithmic transformation for ASVs and Faith’s phylogenetic diversity. We used linear models to test whether the alpha diversity values differed between biome and localities from what was expected by randomness and tested the effects of sex, location, year, and biome.

To calculate beta diversity measures, we first filtered out all singletons from our data set. We implemented a prevalence filter whereby any ASV not present in at least 7% of the samples was pruned out of the database. We first calculated the dissimilarity matrix by using the weighted UniFrac and unweighted UniFrac indexes (54, 55). We constructed an ordination and plotted the data by using the locality or the year as the grouping variable. The weighted UniFrac incorporated both the taxonomic richness and the specific abundance of a particular ASV; hence, the obtained metrics represented differences between the microbiome’s most abundant components (56). The unweighted UniFrac does not consider the abundance of an ASV; thus, the obtained metrics represent differences in the microbiome’s overall taxonomic breadth and are driven mainly by rare taxa (57). Using these ordinations, we calculated the centroid for each group and plotted them together with all the data points and the 95% ellipses plus the explored biome and locality patterns for the whole data set. We calculated Cohen’s D and the 95% confidence intervals to measure the effect sizes between treatments by using the R package “*compute.es*” (58). If the confidence interval included the “zero,” the differences were considered nonsignificant. We used the “*betadisper*” function in the “*vegan*” package in R in order to calculate the data dispersion (PERMDISP2) around the centroid for each year (59). The data dispersion is used as a proxy for variability within the groups. We tested for differences in beta diversity measures between groups by using a permutational multivariate analysis of variance using distance matrices (PERMANOVA [60]) implementing the Adonis function in the *vegan* package (61).

Finally, to test for the differential abundance of specific ASVs, we employed an analysis of compositions of microbiomes, namely, ANCOM v.2.1 (62). This method allowed us to distinguish between sampling and structural zeroes in the data (see Kaul et al. [63] for details). We created a volcano plot mapping by family and genus for the differentially abundant taxa. In these plots, the more negative the value, the more differentially abundant taxa are on the first site of the comparison, while a strong positive value is an indication of that taxon being more differentially abundant on the other site.
