# Supplementary material for: A Faithful Gut: Core Features of Gastrointestinal Microbiota of Long-Distance Migratory Bats Remain Stable despite Dietary Shifts Driving Differences in Specific Bacterial Taxa
Source: Microbiol Spectr. 2021 Nov 24;9(3):e01525-21. doi: 10.1128/Spectrum.01525-21 (PMC8612142; doi:10.1128/Spectrum.01525-21)
Supplement: SUPPLEMENTAL FILE 1 — Supplemental material. Download SPECTRUM01525-21_Supp_1_seq12.pdf, PDF file, 2.0 MB [file spectrum01525-21_supp_1_seq12.pdf]

Figure S1

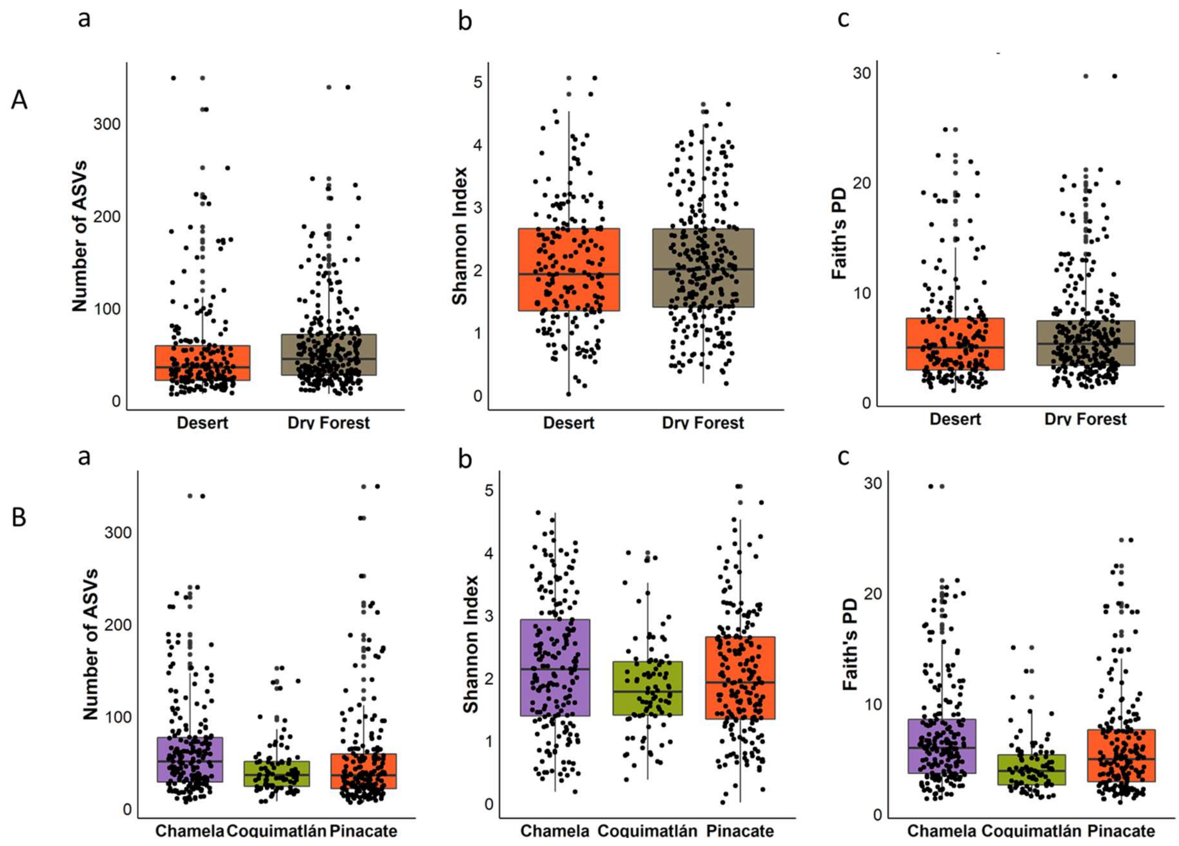

Figure S1. Gut microbial alpha diversity measures of female bats between 2015-2017. A) Grouped by biome, B) Grouped by locality. (a) number of ASVs, (b) Shannon Index, (c) Faith's PD.

S. Table 1. Geographical coordinates, and extra information for all sampling sites.

Biome	Locality	Other names	Municipality	Lat	Long	Ecosystem	Foraging area	Other species
**Dry Forest**	Chamela	Isla San Panchito, Isla San Agustín	La Huerta, Jalisco	19.534395	-105.088262	Island cave off the coast of Xametla	autumn and winter	Pteronotus spp.
	Coquimatlan	La Fábrica	Coquimatlán, Colima	19.151177	-103.834796	Cave with guano collection activity	year-long?	Pteronotus spp.
**Desert**	Pinacate	Cueva del Pinacate	Sonoyta, Sonora	31.647611	-113.480889	Collapse lava tunnel, Sonoran Desert	May to August	none

S. Table 2. Provenance of collected samples

Biome	Locality	2015		2016		2017		total
		Male	Female	Male	Female	Male	Female	
**Dry Forest**	Chamela	-	-	51	69	30	65	**307**
	Coquimatlan	-	-	-	-	60	32	
**Desert**	Pinacate	0	50	0	82	0	81	**213**

S. Table 3. Linear models for Alpha diversite measurements by locality and biome.

Log(FPD) by Biome

Coefficients	Estimate	Std. Error	t value	Pr(>|t|)	
(Intercept)	187.153	0.08969	20.866	< 2e-16	***
year2016	-0.18719	0.10875	-1.721	0.086114	.
year2017	-0.54347	0.10814	-5.026	8.08e-07	***
BiomeDry Forest	0.25217	0.07018	3.593	0.000374	***

Model	Df	Sum Sq	Mean Sq	F value	Pr(>F)	
year	2	10.998	54.990	14.859	0,000645	***
Biome	1	4.779	47.787	12.913	0.0003739	***
Residuals	345	127.677	0.3701			

Log(ASV) by Biome

Coefficients	Estimate	Std. Error	t value	Pr(>|t|)	
(Intercept)	54.761	7.282	7520	4.78e-13	***
year2016	8.792	8.829	0.996	0.3200	
year2017	-13.610	8780	-1550	0.1220	
BiomeDry Forest	11.440	5.697	2008	0.0454	*

Model	Df	Sum Sq	Mean Sq	F value	Pr(>F)	
year	2	34343	17171.7	70.397	0.001008	**
Biome	1	9836	9835.6	40.322	0.045419	*
Residuals	345	841549	2439.3			

Sqrt(SW) by Biome

Coefficients	Estimate	Std. Error	t value	Pr(>|t|)	
(Intercept)	137.686	0.05049	27.272	<2e-16	***
year2016	0.05031	0.06121	0.822	0.412	
year2017	-0.04395	0.06087	-0.722	0.471	
BiomeDry Forest	0.06229	0.03950	1577	0.116	

Model	Df	Sum Sq	Mean Sq	F value	Pr(>F)	
year	2	0.622	0.31104	26.529	0.07188	.
Biome	1	0.292	0.29156	24.867	0.11573	
Residuals	345	40.450	0.11725			

Log(FPD) by Locality

Coefficients	Estimate	Std. Error	t value	Pr(>|t|)	
(Intercept)	2.17777	0.11366	19.161	< 2e-16	***
LocalityCoquimatlan	-0.32232	0.08689	-3.710	0.000232	***
LocalityPinacate	-0.30624	0.07107	-4.309	1.99e-05	***
sexM	-0.06689	0.07279	-0.919	0.358534	
year2016	-0.26505	0.10669	-2.484	0.013319	*
year2017	-0.46966	0.10616	-4.424	1.20e-05	***

Model	Df	Sum Sq	Mean Sq	F value	Pr(>F)	
Locality	2	13.275	66.373	18.341	2,11E-05	***
sex	1	0.157	0.1571	0.434	0.5103	
year	2	8.441	42.207	11.663	1,13E-02	***
Residuals	481	174.063	0.3619			

Log (ASV) by Locality

Coefficients	Estimate	SE	t value	Pr(>|t|)	
(Intercept)	68.290	9.288	7.352	8.44e-13	***
LocalityCoquimatlan	-13.499	7.101	-1.901	0.0579	.
LocalityPinacate	-13.529	5.808	-2.329	0.0202	*
sexM	-2.705	5.948	-0.455	0.6494	
year2016	4.508	8.719	0.517	0.6054	
year2017	-9.548	8.676	-1.101	0.2717	

Model	Df	Sum Sq	Mean Sq	F value	Pr(>F)	
Locality	2	34305	17152.4	70.973	0.0009168	***
sex	1	133	133.4	0.0552	0.8143290	
year	2	17340	8670.2	35.876	0.0284087	*
Residuals	481	1162451	2416.7			

Sqrt(SW) by Locality

Coefficients	Estimate	Std. Error	t value	Pr(>|t|)	
(Intercept)	1.44667	0.06705	21.576	<2e-16	***
LocalityCoquimatlan	-0.06507	0.05126	-1.269	0.2049	
LocalityPinacate	-0.06981	0.04193	-1.665	0.0965	.
sexM	-0.05493	0.04294	-1.279	0.2014	
year2016	0.01480	0.06294	0.235	0.8141	
year2017	-0.01029	0.06263	-0.164	0.8695	

Model	Df	Sum Sq	Mean Sq	F value	Pr(>F)	
Locality	2	0.587	0.293645	23.317	0.09823	.
sex	1	0.190	0.190224	15.105	0.21967	
year	2	0.055	0.027298	0.2168	0.80520	
Residuals	481	60.575	0.125936			

S Table 4. Permanova model and coefficients for Unweighted and Weighted Unifrac.

a. Model for UnWeighted UniFrac

	Df	SumsOfSqs	MeanSqs	F.Model	R2	Pr(>F)
Locality	2	13.654	68.272	42.035	0.14405	1,00E-04
year	2	1.956	0.9778	6.020	0.02063	1,00E-04
Locality:year	1	1055	10553	6.498	0.01113	1,00E-04
Residuals	481	78.123	0.1624		0.82418	

b. Model for Weighted UniFrac

	Df	SumsOfSqs	MeanSqs	F.Model	R2	Pr(>F)
Locality	2	11.191	55.954	226.009	0.08309	1,00E-04
year	2	2.549	12.746	51.483	0.01893	1,00E-04
Locality:year	1	1.855	18.551	74.933	0.01377	1,00E-04
Residuals	481	119.083	0.2476		0.88420	

S Table 5. Pairwise comparisons between year-site categories for Weighted and UnWeighted UniFrac.

a. Paiwise comparisons for Weighted UniFrac

Pair	difference	lower	upper	p adj
Chamela 2017-Chamela 2016	-0.005007231	-0.057956917	0.04794245	0.9989988
Pinacate 2015-Chamela 2016	-0.031592825	-0.088459692	0.02527404	0.5474154
Pinacate 2016-Chamela 2016	-0.037382074	-0.087899044	0.01313490	0.2539131
Pinacate 2017-Chamela 2016	0.042740444	-0.007175640	0.09265653	0.1323581
Pinacate 2015-Chamela 2017	-0.026585594	-0.083655826	0.03048464	0.7048539
Pinacate 2016-Chamela 2017	-0.032374842	-0.083120631	0.01837095	0.4046624
Pinacate 2017-Chamela 2017	0.047747676	-0.002399969	0.09789532	0.0705282
Pinacate 2016-Pinacate 2015	-0.005789248	-0.060609924	0.04903143	0.9984551
**Pinacate 2017-Pinacate 2015**	**0.074333269**	**0.020065806**	**0.12860073**	**0.0018965**
**Pinacate 2017-Pinacate 2016**	**0.080122518**	**0.032550663**	**0.12769437**	**0.0000547**

b. Paiwise comparisons for UnWeighted UniFrac

Pair	difference	lower	upper	p adj
Chamela 2017-Chamela 2016	-0.038606718	-0.080495409	0.003281973	0.0869155
Pinacate 2015-Chamela 2016	-0.027397521	-0.072385108	0.017590066	0.4534721
Pinacate 2016-Chamela 2016	0.019436876	-0.020527285	0.059401037	0.6698908
Pinacate 2017-Chamela 2016	0.026749907	-0.012738890	0.066238705	0.3420290
Pinacate 2015-Chamela 2017	0.011209197	-0.033939273	0.056357667	0.9604130
**Pinacate 2016-Chamela 2017**	**0.058043594**	**0.017898414**	**0.098188774**	**0.0008540**
**Pinacate 2017-Chamela 2017**	**0.065356626**	**0.025684640**	**0.105028612**	**0.0000856**
**Pinacate 2016-Pinacate 2015**	**0.046834397**	**0.003465559**	**0.090203234**	**0.0269789**
**Pinacate 2017-Pinacate 2015**	**0.054147429**	**0.011216239**	**0.097078618**	**0.0055135**
Pinacate 2017-Pinacate 2016	0.007313032	-0.030321237	0.044947301	0.9838670
